# Regarding: CXCL10 Secreted by SPRY1‐Deficient Epidermal Keratinocytes Fuels Joint Inflammation in Psoriatic Arthritis via CD14 Signalling

**DOI:** 10.1111/jcmm.70887

**Published:** 2025-10-07

**Authors:** DuJiang Yang, Jiafeng Song, Junjie Chen, Shuang Wang, GuoYou Wang

**Affiliations:** ^1^ The Affiliated Traditional Chinese Medicine Hospital, Southwest Medical University Luzhou People's Republic of China; ^2^ Center for Orthopedic Diseases Research The Affiliated Traditional Chinese Medicine Hospital, Southwest Medical University Luzhou China

Dear Editor,

The recent work by Xu et al. (JCI 186135) provides a transformative perspective on the pathophysiology of psoriatic arthritis (PsA) by elucidating a precise mechanistic link between cutaneous inflammation and articular involvement [1]. Their findings position the SPRY1/CXCL10/CD14 axis as a critical driver of disease, offering a paradigm shift from viewing PsA merely as a comorbid condition to understanding it as a systemically connected process orchestrated by specific molecular cues from the skin.

This study compellingly demonstrates that deficiency of SPRY1 in epidermal keratinocytes is not only a progenitor of psoriatic dermatitis but also a potent instigator of joint pathology. The authors utilise a keratinocyte‐specific Spry1‐knockout mouse model to recapitulate key features of PsA [1]. The central revelation is that these keratinocytes secrete high levels of CXCL10, which acts as a chemotactic signal, mobilising CXCR3+ immune cells to the joints [1]. The subsequent engagement of the CD14 receptor on macrophages, leading to NF‐κB activation and TNF‐α production, represents a significant mechanistic insight [1]. This work aligns with emerging evidence that CXCL10 is among the most promising biomarkers for predicting PsA development in psoriasis patients [2].

The translational implications of these findings are substantial [3]. The demonstration that Cd14‐deficient mice are resistant to CXCL10‐induced arthritis underscores the non‐redundant role of CD14 signalling in this pathway [1]. This is particularly relevant given recent clinical trials showing that CXCL10 levels decrease following effective Tyk2 inhibition with deucravacitinib, and that higher baseline CXCL10 levels predict better treatment response [4]. These parallel findings suggest that CXCL10 might serve both as a predictive biomarker and a therapeutic target.

However, several intriguing questions emerge. First, the precise molecular nature of the CXCL10‐CD14 interaction requires further elucidation. Second, while the role in macrophages is clear, does CD14 signalling in other myeloid cells contribute to the pathology? Third, the relationship between this novel pathway and established cytokine networks in PsA, particularly the IL‐23/IL‐17 axis, needs clarification [5]. Recent research has confirmed that IL‐17A remains a crucial mediator in PsA pathogenesis [6], and the interplay between CXCL10 and IL‐17A signalling merits investigation.

The clinical applications of these discoveries are promising. Targeting the CXCL10/CD14 interface could offer a strategic therapeutic advantage by intercepting the inflammatory signal en route from the skin to the joint. This approach might potentially prevent arthritis development in high‐risk psoriasis patients [2]. The data suggest that circulating CXCL10 levels could help stratify psoriasis patients at the highest risk for developing PsA, enabling preemptive clinical management [7].

In conclusion, Xu et al. have delivered a landmark study that successfully deciphers a key component of the ‘skin‐joint axis’ in PsA [1]. They provide robust preclinical evidence that keratinocyte‐derived CXCL10, fueled by SPRY1 deficiency, propagates joint inflammation via CD14‐mediated innate immune activation. This work not only deepens our understanding of PsA aetiology but also unveils the CXCL10/CD14 signalling pathway as a compelling new therapeutic target worthy of rapid clinical investigation.

## Author Contributions

**DuJiang Yang:** conceptualization, investigation, writing – review and editing, writing – original draft, methodology. **Jiafeng Song:** methodology, validation. **Junjie Chen:** methodology, software. **Shuang Wang:** writing – review and editing, writing – original draft, investigation. **GuoYou Wang:** funding acquisition, investigation.

## Ethics Statement

The authors have nothing to report.

## Consent

The authors have nothing to report.

## Conflicts of Interest

The authors declare no conflicts of interest.

## Data Availability

Data sharing not applicable to this article as no datasets were generated or analysed during the current study.
